# Extracellular Vesicles Delivered a Functional ARG1 Enzyme and Restored Its Activity in a Mouse Model of ARG1-D Resulting in Improved Lifespan

**DOI:** 10.3390/ijms27093785

**Published:** 2026-04-24

**Authors:** Li-En Hsieh, Mafalda Cacciottolo, Michael J. LeClaire, William Morrison, Bailey Murphy, Christy Lau, Kristi Elliott, Linda Marban, Minghao Sun

**Affiliations:** Capricor Therapeutics, Inc., 10865 Road to the Cure, San Diego, CA 92121, USA; lhsieh@capricor.com (L.-E.H.); mcacciottolo@capricor.com (M.C.); mleclaire@capricor.com (M.J.L.); wmorrison@capricor.com (W.M.); bmurphy@capricor.com (B.M.); clau@capricor.com (C.L.); kelliott@capricor.com (K.E.); lmarban@capricor.com (L.M.)

**Keywords:** extracellular vesicles (EVs), arginase 1 deficiency, hyperargininemia, enzyme replacement therapy

## Abstract

Arginase 1 (ARG1) deficiency (ARG1-D) is a rare genetic disorder due to loss of ARG1, the final enzyme in the urea cycle. ARG1-D hepatocytes are impaired in converting arginine into urea, resulting in elevated peripheral arginine and ammonia, which leads to progressive neurological symptoms. Current therapeutic strategies mainly focus on managing plasma arginine and ammonia level, but long-term outcomes remain poor. While no approved treatment specific for ARG1-D is available in the United States, a recombinant protein-based enzyme replacement therapy is available in Europe. Recently, extracellular vesicles (EVs) are emerging as a powerful therapeutic vehicle. By using Capricor’s StealthX^TM^ platform, EVs were engineered to express human ARG1 on their surface or encapsulated within. Regardless of their localization on the EV membrane, nanograms of ARG1 carried by EVs were biologically active and able to convert arginine into urea as potent as micrograms of human recombinant ARG1 (rHuArg1). Furthermore, ARG1-encapsulating EVs (STX-Arg1-in) were able to deliver ARG1 intracellularly but not EVs carrying ARG1 on their surface or rHuArg1. STX-Arg1-in EVs were further evaluated in a series of in vivo studies, and the results showed that STX-Arg1-in EVs were non-toxic and able to restore arginase activities in the liver of Arg1^−/−^ mice, which led to a lowered plasma arginine concentration similar to that in wild-type mice. Most importantly, Arg1-in EVs expanded the lifespan of the lethal neonatal Arg1 deficiency mouse model. Taken together, our data suggested StealthX^TM^-engineered STX-Arg1-in EVs have a better safety profile due to the extremely low dosage and have great potential as a novel enzyme replacement strategy for patients suffering from ARG1-D. Significance statement: Intracellular delivery of recombinant protein and improved llifespanare endpoints of successful enzyme replacement therapy for the treatment of ARG1-D. Using the StealthX platform, a fully functional ARG1 enzyme was engineered to be carried inside of the extracellular vesicles, which allowed for the intracellular delivery of ARG1 protein in vitro and in vivo, with an improvement of lifespan in a lethal neonatal mouse model of Arg1 deficiency. More importantly, no toxicity was observed, and efficacy was achieved with a low dose, setting the base for an improved therapeutic approach.

## 1. Introduction

Arginase 1 (ARG1) deficiency (ARG1-D) is a rare autosomal recessive genetic disorder that occurs in approximately 2.8 cases per million births with the population prevalence at approximately 1.4 cases per million people worldwide [1,2]. ARG1-D is one of the diseases among the urea cycle disorders (UCDs) and is caused by the mutations or deletions of the ARG1 gene located on chromosome 6 (6q23), which leads to a lack of ARG1 protein expression in the patients [3]. ARG1 is the last enzyme in the urea cycle to catalyze the conversion of arginine into ornithine and urea [3]. The urea cycle mainly takes place in the hepatocytes in the liver and is the major pathway to detoxify ammonia in mammals [3]. Patients with ARG1-D usually display symptoms in their late infancy to pre-school age [4]. With the disease progression, patients would show spasticity mainly in their lower limbs, intellectual disability, motor deficits, seizures, developmental delays/growth deficiency, and other neurological symptoms, which impact the quality of life of the patients and their parents/caregivers [1,3,5]. The standard therapeutic interventions mainly focus on controlling the plasma arginine through dietary protein restrictions, supplementing arginine-free essential amino acids, and the administration of nitrogen scavengers to control the plasma ammonia level [1,3,5,6]. Over the past two decades, different approaches for ARG1-D-specific therapies have been evaluated, such as recombinant human ARG1 protein [7], adeno-associated virus (AAV)-based therapies [8,9,10,11], and lipid-nanoparticle-based mRNA therapy [12]. Despite the promising data in the preclinical stage, none of these strategies have produced a valuable therapy but the pegylated human ARG1, which was recently approved in the European Union and United Kingdom (https://www.ema.europa.eu/ Accessed on 2 January 2025). Although the pegylated human ARG1 treatment resulted in improved plasma arginine levels in the preclinical studies [7] and clinical trials [13,14], this approach showed no improvement of lifespans in ARG1-D mouse models [7], presumably due to the lack of liver-specific delivery.

For the past decade, extracellular vesicles (EVs) have drawn significant attention for their potential applications in both diagnostics and therapeutics [15,16]. EVs are nano-sized vesicles naturally secreted by almost all cell types [17,18], ~50–150 nm in size, formed from the inward budding of the endosome membranes, functioning as a “messenger” between cells, and involved in different physiological and pathological conditions [19]. EVs showed low immunogenicity with good biocompatibility and a good safety profile, which are the key features for ideal nanomedicine [15,19,20,21,22]. Numerous studies have demonstrated the capability of loading different therapeutic cargos, including protein, small RNA, and small molecules, and many of them are being evaluated in the early to late stages of clinical trials [19,20,22,23]. However, so far, there is no approved EV-based therapy according to the US Food and Drug Administration.

The goal of the present study is to develop an enzyme replacement therapy for ARG1-D using our StealthX^TM^ exosome-based platform. We successfully engineered 293F cells to express a functional ARG1 enzyme onto the EVs (STX-Arg1) membrane, which resulted in ARG1 delivery to the cells while retaining its enzymatic activity. Our data suggested that the ARG1 EVs were safe and capable of increasing ARG1 activity in the liver and decreasing the levels of circulating arginine at the same time in a wild-type mouse model with a nanogram dosage. Furthermore, the treatment of neonatal Arg1 deficiency in a mouse model with STX-Arg1 EVs resulted in an increased lifespan because of the active delivery of ARG1 into the liver and reduced circulating arginine. The results presented here demonstrate the efficacy and safety of our STX-Arg1 EVs and show tremendous potential as enzyme replacement therapy for the treatment of ARG1-D.

## 2. Results

### 2.1. ARG1 Protein Expression on Engineered Cells and EVs

**Figure 1:**
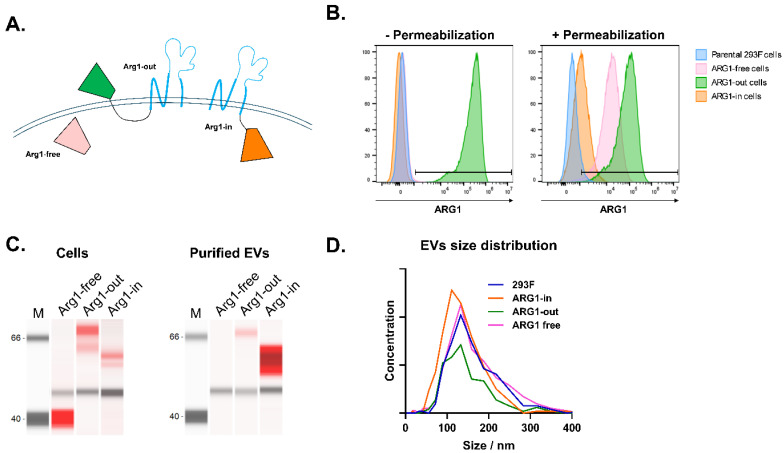
Characterization of StealthX^TM^-engineered cells and EV. (**A**) A scheme showing the design of the expression of the ARG1 protein with different anchor designs and orientations on the cell surface (pink: free ARG1; green: STX-ARG1-out; orange: STX-ARG1-in; blue: tetraspanin CD9). (**B**) The flow cytometry analysis of the ARG1 protein expression on different ARG1 cell lines. Parental 293F cells served as the control, and no expression of ARG1 protein was detected. Extracellular ARG1 expression was only detected on Arg1-out cells (− permeabilization). ARG1 protein expression on Arg1-free and Arg1-in cells can only be detected after fixation and permeabilization of the cells (+ permeabilization) (pink: free ARG1; green: STX-Arg1-out; orange: STX-Arg1-in; blue: parental cell line). (**C**) ARG1 expressions on cells and EVs were confirmed by Simple Western blotting. Near-infrared red: ARG1 (STX-Arg1 in: ~60 kDa; STX-Arg1-out: ~70 kDa; free Arg1: ~40 kDa). Chemiluminescence: actin, used as loading control ~50 kDa. (**D**) The size distribution of 293F and STX-Arg1 EVs by ZetaView nanoparticle tracking analysis (NTA).

Using Capricor’s proprietary StealthX^TM^ platform, 293F cells were engineered to express the ARG1 protein on the EV membrane. Because the localization of the ARG1 protein on the EV membrane could affect delivery and its activity, three different constructs were designed to allow for the expression of ARG1 protein as (1) a free form (Arg1-free), (2) on the cell surface by linking to the N-terminal of the tetraspanin CD9 (STX-Arg1-out), or (3) tethered to the C-terminal of the CD9 (STX-Arg1-in) (Figure 1A). ARG1 protein expression was confirmed by flow cytometry (Figure 1B) and Jess Western blotting (Figure 1C). As shown in Figure 1B, the localization of ARG1 matched the original design (as illustrated in Figure 1A). The enrichment of exosome markers (CD9, CD81) and the absence of cellular markers (calnexin, GM130, Hsp60) was confirmed by Jess Western blotting (Supplementary Figure S1).

Next, EVs carrying the ARG1 protein (STX-Arg1) were produced and purified from the culture supernatants. The concentrations of the purified EVs ranged between 0.96 × 10^12^ and 3.0 × 10^12^ particles/mL, with an average diameter of 120.3–129.5 nm and a polydispersity index (PDI) of 0.133–0.151 (Figure 1D, Table 1). Enrichment of ARG1 protein in the EVs preparations was confirmed by Jess Simple Western analysis for both STX-Arg1-in and STX-Arg1-out, but not for Arg1-free EVs (Figure 1C). Furthermore, the ARG1 protein was carried by 1 × 10^11^ EV/mL of Arg1-out EVs, and Arg1-in EVs were 7.0 ± 0.5 and 8.7 ± 0.2 ng/mL, with the arginase activities at 51.1 ± 1.3 and 226.4 ± 5.7 U/L, respectively (Table 1). Arg1-free EVs, on the other hand, showed minimal detection of the ARG1 protein by Simple Western protein analyses (Figure 1C, right panel), with 3.7 ± 0.4 ng/1 × 10^11^/mL EVs and 6.0 ± 0.0 U/L arginase activity (Table 1). Due to the low arginase activities from the Arg1-free EVs, only Arg1-out and Arg1-in were further studied for the in vitro delivery of the ARG1 protein.

### 2.2. In Vitro Delivery of ARG1 by Arg1-in EVs

**Figure 2:**
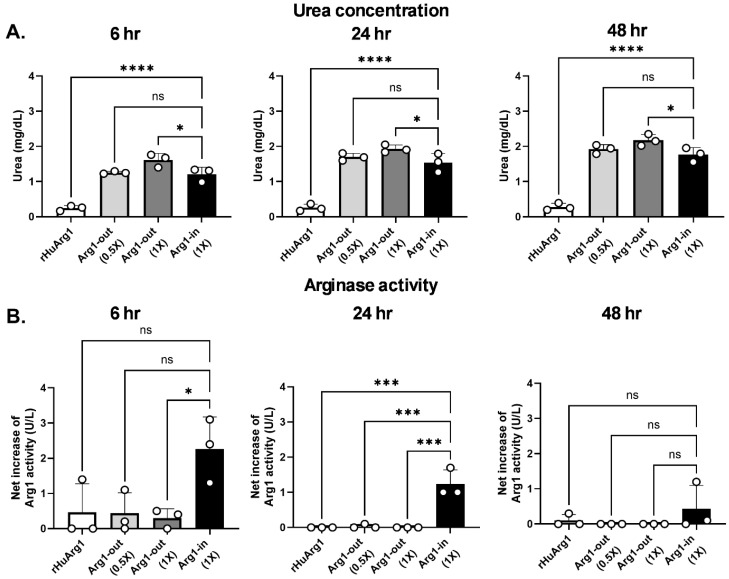
The evaluation of intracellular ARG1 delivery by ARG1 EVs in vitro. HepG2 cells were treated with 1X (1 × 10^11^; 8.7 ng of ARG1) Arg1-in EV, 0.5X (1 × 10^11^; 4.5 ng ARG1) or 1X (1.96 × 10^11^; 8.7 ng of ARG1) Arg1-out EV, or 8.7 ng of rHuArg1 protein for 6, 24, and 48 h. Culture supernatants and cell lysates were collected separately to evaluate the enzymatic activity of ARG1. (**A**) Urea concentrations in the culture supernatant. Urea productions in both Arg1-in and Arg1-out EV-treated cell cultures were time-dependent. ARG1 carried by EVs was more efficient in converting arginine into urea compared to the same concentration of rHuArg1. (**B**) Arginase activities in the cell lysates. Unlike rHuArg1 and Arg1-out EVs that failed to achieve the intracellular delivery of ARG1, increased arginase activities can be readily detected in the HepG2 cells 6 h after Arg1-in EV treatment and gradually decreased overtime. N = 3 independent experiments. Data shown as mean ± STDEV. Data was analyzed using an ordinary one-way ANOVA with Tukey’s multiple comparison test. A *p* value < 0.05 was considered to be statistically significant. ns: not significant. *: *p* < 0.05. ***: *p* < 0.001. ****: *p* < 0.0001.

To evaluate the enzyme activity of STX-Arg1 EVs and test their ability to deliver functional ARG1 protein into the cells, EVs carrying ARG1 anchored on their membrane (STX-Arg1-out and STX-Arg1-in) were compared to the recombinant human ARG1 protein (rHuArg1) for the catalyzation of arginine and the intracellular delivery of arginase activities. A hepatocellular carcinoma cell line, HepG2 cells, was treated with 1 × 10^11^ Arg1-in EVs (carrying 8.7 ng of ARG1 protein; 1X), 1 × 10^11^ (0.5X) or 1.96 × 10^11^ (1X) Arg-out EVs (carrying 4.5 or 8.7 ng of ARG1 protein), or 8.7 ng rHuARG1 for 6, 24, and 48 h (hr). Urea concentrations in the culture supernatants and arginase activities in the cell lysates were measured. The results showed that neither the urea concentration (Figure 2A) nor the intracellular arginase activity (Figure 2B) were affected by the treatment of 8.7 ng rHuArg1 protein across the time points studied. In contrast, 1 × 10^11^ of both STX-Arg1-out and STX-Arg1-in EVs showed a significant increase in the urea concentration in the culture supernatants in a time-dependent manner (Figure 2A). The 1 × 10^11^ STX-Arg1-out EVs, which carried ~50% less ARG1 protein compared with rHuArg1 protein control, showed similar urea production compared to 1 × 10^11^ STX-Arg1-in EVs. In addition, the 1.96 × 10^11^ STX-Arg1-out EVs (8.7 ng ARG1 protein) showed significantly higher urea production compared to STX-Arg1-in at 6 (*p* = 0.0103), 24 (*p* = 0.0407), and 48 (*p* = 0.0159) h after treatment, suggesting that the ARG1 protein carried outside of the EVs can catalyze arginine more efficiently. In contrast, STX-Arg1-out EVs had limited intracellular delivery of the ARG1 protein, similarly to the rHuArg1 (Figure 2B). STX-Arg1-out-treated HepG2 cells showed only a limited increase in arginase activity 6 h after treatment and the arginase activity was undetectable afterward, despite the dose used (Figure 2B). On the other hand, treatment with STX-Arg1-in EVs increased intracellular arginase activities in the HepG2 cells, and the level was higher than both rHuArg1 and the two concentrations of Arg1-out EVs tested. Additionally, after STX-Arg1-in treatment, arginase activities remain elevated 48 h after treatment (Figure 2B). As control, urea production detected in the culture supernatant from the culture media treated with rHuArg1 or STX-Arg1 EVs in the absence of cells was tested and reported in Supplementary Figure S2A, showing bioactivity. To exclude the possibility that EVs are interacting with the plasma membrane of HepG2 cells and triggering intracellular signals to promote specific functions, HepG2 cells were treated with either 293F parental EVs, STX-Arg1-in EVs or rHuArg1. As shown in Supplementary Figure S2B, 293F EVs did not trigger any Arg1 activities in cells, confirming that the resulting effect is due to the Arg1 delivered by STX-engineered EVs. Overall, the data showed that although the ARG1 protein displayed on the surface of EVs (Arg1-out) was more potent in catalyzing arginine, only the ARG1 protein encapsulated inside the EVs (Arg1-in) could deliver the ARG1 protein into the cells and maintain its enzymatic activity for longer periods of time.

### 2.3. The Potency of Arg1-in EVs

**Figure 3:**
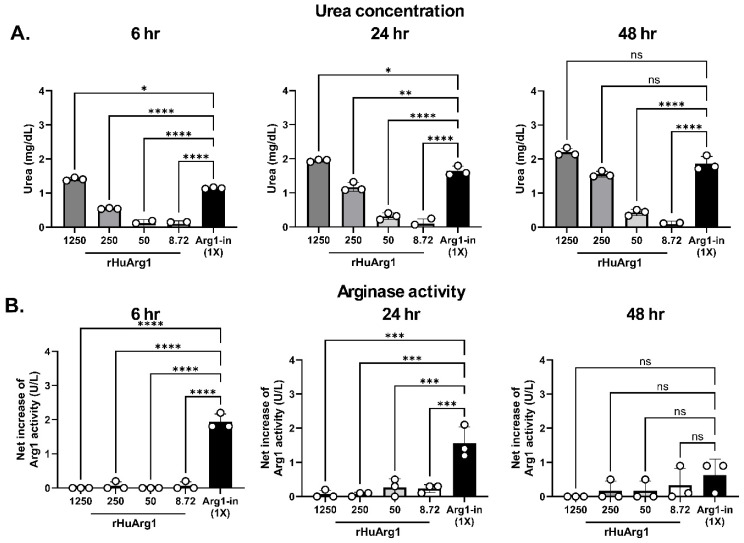
The potency of Arg1-in EVs compared to rHuArg1. HepG2 cells were treated with 1 × 10^11^ (8.7 ng ARG1) Arg1-in EVs or with a scalar dose of rHuArg1 (8.7, 50, 250, 1250 ng), and culture supernatants and cell lysates were harvested separately 6, 24, and 48 h after treatment to evaluate the potency of the Arg1-in EVs. (**A**) Urea concentrations in the culture supernatant. Urea concentrations from the cell cultures treated with Arg1-in EVs carrying 8.7 ng ARG1 were comparable to the cells treated with 1250 ng of rHuArg1 and were significantly higher than the ones from rHuArg1 at 8.7, 50 and 250 ng-treated cells across the time points tested. (**B**) Arginase activities in the cell lysates. Arg1-in EV treatment significantly increased the intracellular arginase activity in HepG2 cells. Arginase activity in rHuArg1-treated cells remained at the background level despite the highest concentration tested being 143.7-fold higher than the Arg1-in EVs. N = 3 independent experiments. Data shown as mean ± STDEV. Data was analyzed using an ordinary one-way ANOVA with Tukey’s multiple comparison test. A *p* value < 0.05 was considered statistically significant. ns: not significant. *: *p* < 0.05. **: *p* < 0.01. ***: *p* < 0.001. ****: *p* < 0.0001.

Since STX-Arg1-in EVs demonstrated a better intracellular delivery of the functional ARG1 protein, additional studies were performed to elucidate the potency of ARG1 EVs. Firstly, a dose response study was performed to identify the optimal dose (EV amount, ARG1 concentration) for STX-Arg1-EVs: as shown in Supplementary Figure S3, a dose of 1 × 10^11^ EVs resulted in higher ARG1 activity and urea production; therefore, it was chosen for subsequent studies. Next, STX-Arg1-in EVs was compared to increasing doses of rHuArg1: urea levels and the intracellular delivery of ARG1 protein were used as endpoints to assess their respective potency. HepG2 cells were treated with 1 × 10^11^ STX-Arg1-in EVs (8.7 ng of ARG1), or 8.7, 50, 250, or 1250 ng of rHuARG1, and urea production and arginase activities were monitored. Consistent with previous data, urea concentrations in the culture supernatants from HepG2 cells treated with STX-ARG1-in EVs were detected 6 h post-treatment and gradually increased afterward (Figure 3A). Similar trends of the increase in urea concentration were also observed in the culture supernatant from HepG2 cells treated with rHuArg1 at 1250, 250, and 50 ng/well, but not 8.7 ng/well (Figure 3A). STX-Arg1-in EVs produced similar levels of urea compared to 1250 ng rHuArg1, and significantly higher levels than 8.7 ng (*p* < 0.0001), 50 ng (*p* < 0.0001), and 250 ng (*p* < 0.05 for 6 and 24 h timepoints) of rHuArg1. Intracellular arginase activity was similarly affected: STX-Arg1-in EVs-treated HepG2 cells showed the highest increase in arginase activities 6 h after treatment, with a decline by the 48 h timepoint (Figure 3B). In contrast, rHuArg1 showed no increase in the arginase activity across timepoints regardless of the concentration (Figure 3B), indicating that STX-Arg1-in had better cell uptake compared with rHuArg1. Overall, STX-Arg1-in EVs outperformed rHuArg1 due to efficient intracellular delivery with higher enzymatic activity at a lower dose.

### 2.4. Biodistribution of STX-Arg1 EVs In Vivo

**Figure 4:**
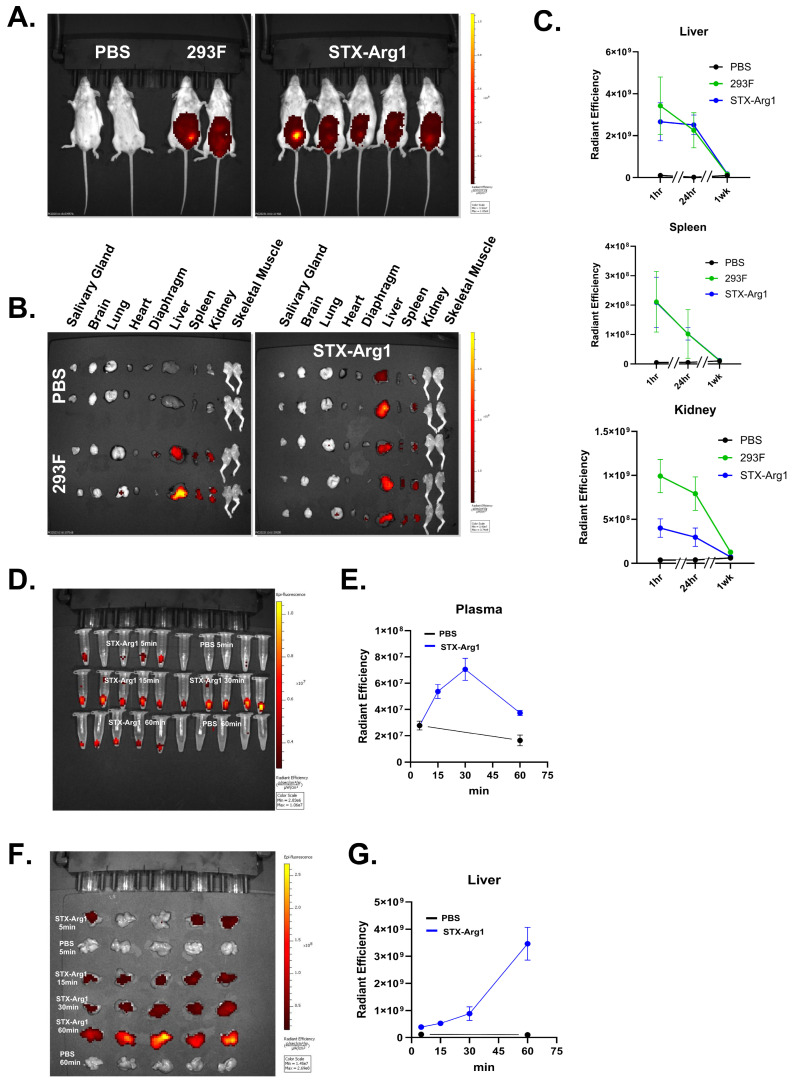
Biodistribution of STX-Arg1 EVs in vivo. The biodistribution of STX-Arg1-in EVs was evaluated in vivo by IVIS imaging. (**A**) A representative IVIS image of PBS control mice, 293F EVs, STX-Arg1-in EVs of injected mice. (**B**) A representative IVIS image of peripheral tissues collected from PBS control, 293F EVs and STX-Arg1-in EV-injected mice. (**C**) The quantification of EVs in tissues 1 h, 24 h and 1 week after injection. (**D**) An IVIS image of plasma collected from PBS and STX-Arg1-in EV-injected mice at different time points. (**E**) The quantification of STX-Arg1-in EV signal in plasma (from (**E**)). The black line is PBS-injected mice; the blue line is STX-Arg1-in EV mice. (**F**) An IVIS image of livers collected from PBS- and STX-Arg1-in EV-injected mice at different time points. (**G**) The quantification of STX-Arg1-in EV signal in plasma (from (**G**)). The black line is PBS injected mice; the blue line is STX-Arg1-in EV mice. N = 5 mice per experimental treatment.

To address the biodistribution of STX-Arg1 EVs, wild-type mice received labeled STX-Arg1-in EVs, and their distribution was analyzed on an IVIS imager (PerkinElmer, Waltham, MA, USA) (Figure 4A–C). The distribution pattern of the STX-Arg1-in EVs resembled the parental 293F EVs. After injection, STX-Arg1-in EVs, similarly to the parental 293F EVs, rapidly distributed to the liver, the spleen and the kidney. After 24 h, the kidney signal increased, indicating clearance. One week (wk) after injection, the STX EVs signal was cleared from the liver, and a minimal signal was recorded in the kidney and the bladder, indicating complete clearance (Figure 4C and Figure S4). The data showed that the biodistribution of STX-Arg1-in EVs was not altered by the expression of the ARG1 protein and aligned with published data on EV distribution [24].

To evaluate the pharmacokinetic of STX-Arg1-in EVs, blood was collected at 1 h and 24 h to evaluate the blood retention of ARG1 EVs. As shown in Figure 4, STX-Arg1-in EVs were detected in blood 1 h after injection, mainly in plasma. No signal was detected in the cellular fraction. The EV-associated signal was completely cleared from circulation by 24 h post-injection (Supplementary Figure S5).

In a separate experiment, STX-Arg1-in EV pharmacodynamic was evaluated at shorter timepoints (Figure 4D–G). The blood and the liver were collected from mice receiving either PBS or labeled STX-Arg1-in EVs at 5, 15, 30, 60 min (min) post-injection. STX-Arg1-in EV-associated signal were quantified and analyzed. As shown in Figure 4D–G, after intraperitoneal injection, STX-Arg1-in EVs rapidly distributed to the circulation, with a peak at 30 min. The STX-Arg1-in EVs were removed from the circulation and distributed to the liver in the following 30 min (Figure 4D–G). This data aligns with the PD/PK of other EVs [24].

### 2.5. In Vivo Toxicity Using Wild-Type Mice

**Figure 5:**
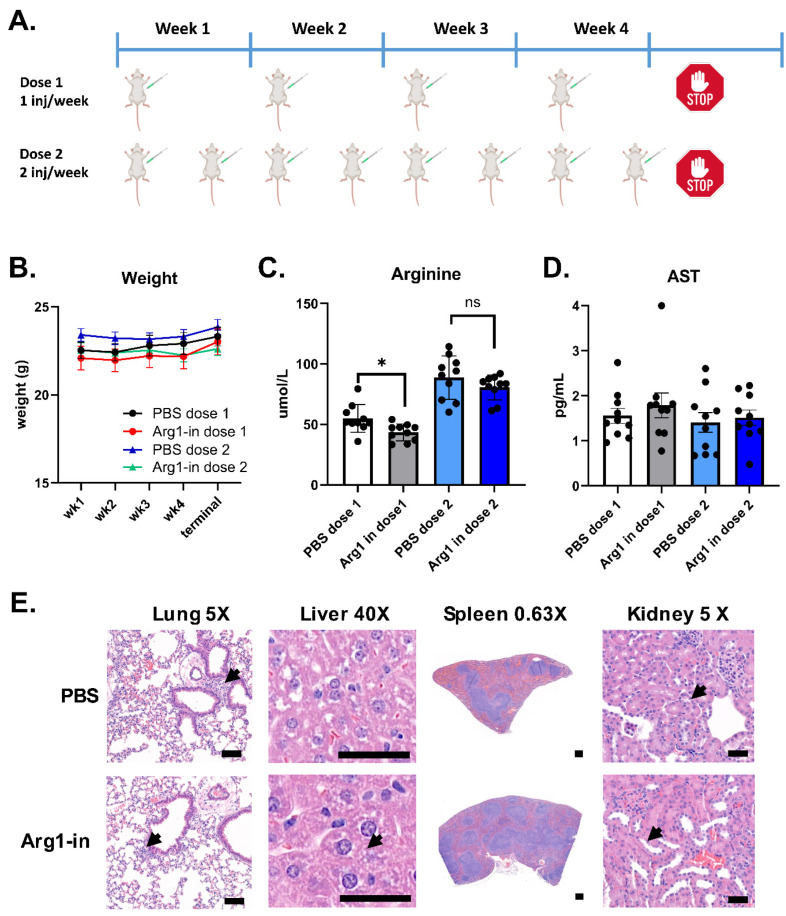
In vivo toxicity of STX-Arg1-in EV injection in wild-type mice. (**A**) A schematic representation of the dosing schedule. (**B**) Weight was monitored weekly. (**C**,**D**) The percentage of change in circulating arginine in mice receiving STX-Arg1-in EVs, compared to PBS group. (**E**) Representative images of H&E-stained tissues from PBS- and STX-Arg1-in-treated mice. Arrows indicate alterations. Scale bar: 50 µm for the lung, the liver and the kidney; 100 µm for spleen. N = 10 mice per experimental group. Data shown as mean ± SEM. 2-tailed *t*-test, ns: not significant, *: *p* < 0.05, N = 10 mice per experimental treatment.

In vivo functionality and toxicity of STX-Arg1-in EVs was first addressed in wild-type mice. Age-matched 8–10 wk old BALB/C mice were assigned to either the control group (receiving vehicle PBS) or STX-Arg1-in EVs at the max calculated dose allowed by the manufacture yield of 0.012 mg/kg. Two dosing schedules were implemented: one cohort received one i.p. injection per week, and one cohort received two i.p. injections per week (Figure 5A). Weight was monitored weekly, and regardless of the dosing schedule, no significant changes in weight were recorded across treatments (Figure 5B). The levels of circulating arginine were quantified by ELISA after terminal blood collection. Lower levels of arginine were observed in the blood of mice receiving one i.p. injection of STX-Arg1-in EVs per week compared to PBS (Figure 5C). A trend of a decrease in arginine concentrations, although not statistically significant, was observed in mice receiving 2 i.p. injections of STX-Arg1 per week (Figure 5C). No changes in AST levels were observed in either dosing group (Figure 5D). Peripheral tissues (see Materials and Methods) were collected for a histology evaluation. At the more frequent dosing schedule, few microscopic alterations were observed, which included minimal to mild leukocyte infiltrates around the airway across all treatments in the lung, 1 µm or smaller cytoplasmic indistinct vacuoles in the liver, minimal to no lymphoid hyperplasia across all treatments in the spleen, and mildly dilated renal tubules in the kidney (Figure 5E). All findings were defined as not adverse, suggesting a safe profile of STX-Arg1-in EVs.

### 2.6. In Vivo Delivery of the ARG1 Protein and Efficacy in the Arg1 Knockout Mouse Model

**Figure 6:**
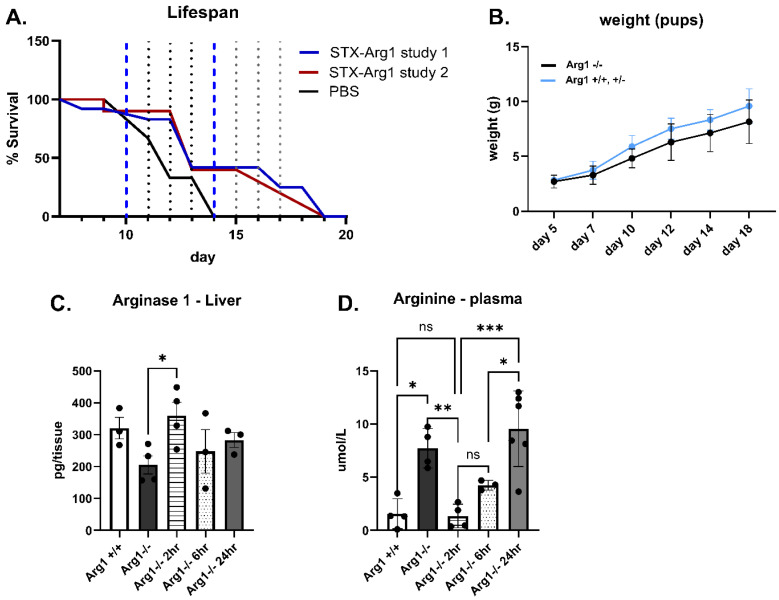
The efficacy of STX-Arg1-in EV therapy in Arg1^−/−^ mice. (**A**) Lifespan analysis. Blue segmented lines represent the window of mortality observed in Arg1^−/−^ mice. Black dots lines identify the single days. Two-way ANOVA showed a *p* = 0002 for treatment effect. (**B**) Weight assessment through treatment. (**C**) ARG1 levels in the livers of naïve wild-type Arg1^+/+^ and knock-out (Arg1^−/−^) mice, and after treatment in Arg1^−/−^ mice. (**D**) Arginine levels in the livers of naïve wild-type (Arg1^+/+^) and knock-out (Arg1^−/−^) mice, and after treatment in Arg1^−/−^ mice. N = 4 Arg1^−/−^ mice assigned to PBS treatment. N = 22 Arg1^−/−^ mice assigned to ARG1 treatment, with 11 mice per each study. One-way ANOVA adjusted for multiple comparisons, ns = not significant, *: *p* < 0.05, **: *p* < 0.01, ***: *p* < 0.005.

To address the therapeutic potential of STX-Arg1-in EVs, the Arg1 knock-out mouse model (Arg1^−/−^) neonatal mice received an i.p. injection of STX-Arg1-in at a maximum dose of 0.03 mg/kg. Repeated dosing resulted in improved survival in Arg1^−/−^ neonatal pups with arginase deficiency (Figure 6A) and weight gain comparable to littermates (Figure 6B): while the Arg1^−/−^ pups receiving vehicle PBS died by day 14 (similarly to what is reported in the literature [7]), 45% of the Arg1^−/−^ pups undergoing STX-Arg1-in EV therapy survived day 14, while 25% survived day 18. A two-way ANOVA analysis showed a *p* = 0.0022, suggesting the effectiveness of the treatment. The positive effect on the lifespan was attributed to the delivery of ARG1 by EVs to the liver cells.

A time-course study was performed on the Arg1^−/−^ mice to address the effect of STX-Arg-in on the lifespan in this model. Arg1^−/−^ pups received the STX-Arg1-in injection and both the blood and the liver were collected 2, 6 and 24 h after the injection. As shown in Figure 6C, STX-Arg1-in EV injection restored liver ARG1 levels in the Arg1^−/−^ pups by 2 h, up to the levels of wild-type littermates, but it was rapidly cleared afterwards, starting at 6 h. Consequently, arginine levels in circulation rapidly declined at 2 h in Arg1^−/−^ undergoing STX-Arg1-in treatment, reaching levels observed in wild-type littermates, and steadily returning to baseline levels by 24 h (Figure 6D). Arg1^−/−^ mice gained weight throughout the study (Figure 6B), but a lethargic phenotype was observed at later life stages, possibly due to hyperammonemia, which is the leading cause of death in this model [7].

## 3. Discussion

Currently, the treatment for ARG1-D mainly relies on the restriction of dietary intake of protein, arginine-free essential amino acid supplements, and nitrogen-scavenging drugs, such as glycerol phenylbutyrate or sodium phenylbutyrate, as needed. Although numerous strategies, including AAV-based gene therapy [8,9,10,11], lipid nanoparticle-based mRNA therapy [12], recombinant ARG1 protein [7], hepatocyte transplantation [25], and stem cell-based therapy [25,26,27], have been evaluated for the treatment for ARG1-D for the past two decades, minimal progress was made in clinical practice. Some approaches were shown to have limited efficacy [7], and the rest were hindered by various challenges, including pre-existing immune responses against gene therapy vectors and immune-mediated toxicities after the administration of AAV vectors [28,29], genotoxicity of AAV-based therapies [30,31], the inability of intracellular delivery of hepatocytes [7], and the safety and adverse effects of synthetic lipid nanocarriers in pediatric cohorts [32]. Thus far, only one disease-specific drug, a cobalt-substituted pegylated recombinant human ARG1 targeting hyperargininemia, was approved until recently in the EU and UK (https://www.ema.europa.eu/, accessed 2 January 2025). It is worth noting that while hyperammonemia is typically not present, the ARG1-D diagnosis is increasingly made by expanded newborn screening. Therapeutic drugs that can directly improve the deficit of the ARG1 protein in ARG1-D remain direly needed.

In this study, we developed an EV-based enzyme replacement therapy that successfully prolonged the lifespan of a lethal neonatal Arg1^−/−^ mouse model. By generating EVs carrying the enzymatic active ARG1 protein on the EV surface or inside of the EV lumen, we showed that the capability in catabolizing arginine by EVs carrying a nanogram of ARG1 were similar to the microgram level of free rHuArg1. We further demonstrated intracellular delivery of the functional ARG1 protein by EVs in vitro, but not the recombinant protein alone. In vivo studies demonstrated that STX-Arg1-in EVs were not toxic. Most importantly, the lifespan of the neonatal Arg1^−/−^ mouse model was extended by the treatment of STX-Arg1-in EV due to the successful delivery of the ARG1 enzyme to the liver, retaining its enzymatic activities and reducing the arginine level in circulation.

EVs were chosen as the vehicles for the cargo delivery of the engineered human ARG1 protein. EVs are cell-derived lipid nanoparticles that serve as a messenger for communication between cells. Due to the nature of the EVs, these cell-derived nanovesicles are considered to be safe, have low immunogenicity, and high biocompatibility [16,21]. Decades-long application of blood transfusion, which contains high concentrations of EVs, further strengthening the safety of this approach. In our previous studies using StealthX^TM^ EV platform, we demonstrated a high safety profile of the STX EVs [33,34]. This was further supported by the current study: no adverse effects, toxicity, or pathological histopathological changes were found in the wild-type mice receiving STX-Arg1-in EVs (Figure 5). The high safety profile allowed us to administrate multiple doses for therapeutic purposes.

The goal of the present study was to develop a novel enzyme replacement therapy for ARG1-D using EV-based approaches to reduce the amount of protein needed for therapeutic efficacy and improve delivery to the liver for the treatment of ARG1-D. As anticipated, the localization of the ARG1 protein on EV membrane affected its functionality. Using our StealthX^TM^ EV-engineering platform, the ARG1 protein was loaded to the EVs through an EV-specific anchor (STX-Arg1-out and STX-Arg1-in) or an anchor-free (Arg1-free) system (Figure 1A). In alignment with previous data, the EV-specific anchor provided a more efficient loading of the ARG1 protein to the EVs, with STX-Arg1-out and STX-Arg1-in EVs showing higher arginase activities compared to the STX-Arg1-free EVs (Figure 1D). To gain a better insight into the potential efficacy, in vitro delivery of the ARG1 protein by ARG1 EVs was studied. Our data showed that nanogram-levels of the ARG1 protein delivered by STX-Arg1-out and STX-Arg1-in EVs had comparable enzyme activities in catalyzing arginine to the microgram level of rHuArg1 (Figure 2A and Figure 3A). As the urea cycle takes place in the hepatocytes in the liver and catabolizes the toxic ammonia into urea [2], the expression of functional ARG1 in the hepatocytes has been suggested to be important to resolve hyperammonemia and prolong the survival of the Arg1 knockout mouse models [7,8,9]. Our data showed that STX-Arg1-in EVs, which have the engineered ARG1 protein encapsulated inside of the EVs, could deliver ARG1 into the cells and retain its function, which was not achievable by either STX-Arg1-out EVs (Figure 2B), or rHuArg1 (Figure 3B). This difference in activity between the two constructs (STX-Arg1-in and STX-Arg1-out) might be due to the localization of the Arg1 protein in respect to the CD9+linker: the linker might be affecting its enzymatic activity. Moreover, it could also be possible that the localization of the enzyme in the extra vesicle space exhausted its enzymatic activity faster, but could still be useful for the clearance of the circulating arginine.

The enrichment of STX-Arg1-in EVs in the liver (Figure 4, Figure 5 and Figure S4) further emphasized the potential of our strategies for the treatment of ARG1-D. The lethal phenotype of the animal models for ARG1-D (Arg1^−/−^ mouse) makes the development of therapeutics challenging. Unlike human ARG1-D, where the disease rarely leads to death caused by the symptoms due to the lack of ARG1 activity directly, both neonatal/congenital and inducible/conditional Arg1-knockout mouse models were lethal [7,27], with a lifespan of 14 days from birth or after induction. The main cause of death of the Arg1-knockout mouse models was considered to be hyperammonemia [7,27], which was less frequent in human ARG1-D [3]. In our study, the therapeutic efficacy of the STX-Arg1-in EVs was evaluated on a neonatal Arg1^−/−^ mouse model, B6.129-Arg1tm1Rki/J (The Jackson Laboratory, Strain # 007741). STX-Arg1-in rapidly delivered ARG1 to the liver, which resulted in a rapid decrease in the circulating plasma arginine. Fast clearance was observed, with a decline 6 h after injection (Figure 6). More importantly, our data showed that the i.p. administration of STX-ARG1-in EVs (at a dose of 0.03 mg/kg of ARG1Arg1) weekly or every 48 h was sufficient to extend the lifespan of the Arg1^−/−^ mice by 45% over day 14, and 25% over day 18 (Figure 5). This result was not achieved by cobalt-substituted pegylated recombinant human ARG1 [7]; the authors suggested that the lack of improvement in survival and the control of hyperammonemia may be caused by the inability of the pegylated recombinant human ARG1 entering the liver, although the treatment was demonstrated to be efficient in controlling arginine concentrations in the circulation. The capability of STX-Arg1-in EVs in controlling blood arginine levels in conjunction with extended lifespan of the Arg1^−/−^ mice strengthens the potential of this strategy as a disease-specific approach in treating ARG1-D.

We are aware of the limitations of the present study. While an improvement in the lifespan was observed after STX-Arg1-in treatment, no long-term survival was achieved. A longer lifespan was achieved by AAV- and LNP-mRNA-based therapies, confirming the need for intracellular delivery of the ARG1 and the constant supply of the enzyme. Further studies with increased amounts of ARG1, new formulations, or engineering strategies to increase the ARG1 half-life are needed to improve the beneficial effects of STX-Arg1-in EV therapy. Additionally, a combination of STX-Arg1-in and STX-Arg1-out could be formulated as a cocktail therapy to combine the potent STX-Arg1-out activity for controlling peripheral arginine levels with the intracellular delivery capability of STX-Arg1-in. Both EV types were biologically active, with no toxic effects, despite the limited data available.

Lastly, the manufacturing of EV-based therapeutics must be considered. Many approaches have been proposed for EV manufacturing [35]: at Capricor, a scalable process has been developed, using tangential filtration and size exclusion chromatography. This approach has been successfully used for the production of clinical doses for vaccine applications and shows scalable capabilities to support therapeutic applications.

Altogether, our data demonstrated the capability of the STX-Arg1-in EVs generated by our StealthX^TM^ platform in the intracellular delivery of the enzymatic active ARG1 protein, which was further translated to increased arginase activity in the liver, relief of hyperargininemia, and most importantly, an extended survival time of a lethal neonatal Arg1-deficiency mouse model. With the safety of EV-based therapeutics, STX-Arg1-in EVs showed a potential in providing a better therapeutic effect in patients suffering from ARG1-D.

## 4. Materials and Methods

### 4.1. Cell Lines

Human embryonic kidney 293 T cells (293T) were purchased from ATCC (Manassas, VA, USA; CRL-3216) and were cultured using Dulbecco’s Modified Eagle Medium (DMEM), high glucose, Glutamax™ containing 10% fetal bovine serum at 37 °C with 5% CO_2_. FreeStyle^TM^ 293F cells (Gibco, Grand Island, NY, USA) were purchased from ThermoFisher Scientific. 293F cells were served as a parental cell line to generate stable cell lines expressing the human ARG1 protein. Parental 293F cells and the engineered 293F cells were cultured in a Multitron incubator (Infors HT, Annapolis Junction, MD, USA) at 37 °C, under an 80% humidified atmosphere with 8% CO_2_ on an orbital shaker platform rotating at 110 rpm.

### 4.2. Lentiviral Vectors

Lentiviral vectors for the expression of human ARG1 (NCBI Reference Sequence: NM_001244438.2) were designed and codon-optimized for the synthesis from Genscript (Piscataway, NJ, USA) together with the two lentiviral packaging plasmids, pMD2.G and psPAX2. Three different designs were utilized: (1) the overexpression of ARG1 (Arg1-free), (2) the extracellular display of ARG1 (Arg1-out) by linking ARG1 with CD9 with a synthetic transmembrane linker at the N-terminal of the ARG1 sequence, and (3) the intracellular packaging of ARG1 (Arg1-in) by linking ARG1 to the C-terminal of the CD9. Production of lentiviral particles for transduction of the cells was performed as previously [33,34]. Briefly, lentiviral particles carrying the gene of interest were generated by transfecting 293T cells with pMD2.G (Genescript), psPAX2 (Genescript) and ARG1 expressing vectors (Genscript) at a ratio of 5:5:1 using Lipofectamine 3000 according to the manufacture’s instruction. Lentiviral particles were collected 48 h after transfection and used for the transduction of 293F cells.

### 4.3. ARG1 EV Production

Suspension Arg1-free, Arg1-out, and Arg1-in cells were cultured in FreeStyle 293 Expression media (Chemical defined, EVs and serum free, ThermoFisher Scientific, Waltham, MA, USA) in a Multitron incubator (Infors HT) at 37 °C under an 80% humidified atmosphere supplied with 8% CO_2_ on an orbital shaker platform. Cell suspension was collected 72 h after seeding, and the cells and cell debris were removed by centrifugation, while microvesicles and other extracellular vesicles larger than ~220 nm were removed by vacuum filtration. Clarified supernatants were processed as previously described [33,34]. Briefly, the supernatant was subjected to concentrating tangential flow filtration (TFF) on an AKTA Flux s instrument (Cytiva, Marlborough, MA, USA) and then subjected to chromatography on an AKTA Avant 25 (Cytiva).

### 4.4. Nanoparticle Tracking Analysis

Size distribution and concentration of purified EVs were determined using ZetaView Nanoparticle Tracking Analysis (Particle Metrix, Mebane, NC, USA) according to manufacturer instructions. EV samples were diluted in 0.1 µm-filtered 1X PBS (Gibco) to fall within the instrument’s optimal operating range to allow the optimal characterization of the EVs. For reproducibility, the following conditions were applied: 1. Number of positions: 11; 2. Resolution: high, 60 frames; 3. sensitivity: 84–87; 4. Shutter: 100; 5. Frame rate: 30 fps; 6. Minimum brightness: 20; 7. Max area: 1000; 8. Min area: 10; 9. Trace length: 15; 10. Laser: 488; 11. Temperature: room temperature; 12. Replicates: three (3) replicates per sample.

### 4.5. Flow Cytometry

ARG1 protein expressions on the cells were detected by standard flow cytometry techniques. The ARG1 protein positioned outside of the cells and EVs (Arg1-out) can be detected by surface staining, while intra-cellular/intra-vesicle ARG1 (Arg1-in) requires membrane permeabilization to allow for its staining/detection. Briefly, for the detection of extracellular ARG1 and CD9, 300,000 cells/well were plated in a 96-well U bottom plate for staining. Cells were incubated at 4 °C for 20 min with 100 µL eBioscience^TM^ Flow Cytometry Staining Buffer (ThermoFisher Scientific) containing anti-human ARG1 antibodies (Biolegend, San Diego, CA, USA, clone 14D2C43, PE) protected from light. For intracellular ARG1 (STX-Arg-in) staining, cells were permeabilized and fixed for 20 min at 4 °C using BD CytoFix/CytoPerm buffer (BD Bioscience, San Diego, CA, USA), followed by incubation at 4 °C for 20 min with 100 µL BD Perm/Wash Buffer (BD bioscience) containing anti-human ARG1 antibodies (Biolegend, clone 14D2C43, PE) protected from light. After staining, cells were washed and re-suspended in 200 µL eBioscience^TM^ Flow Cytometry Staining Buffer (ThermoFisher Scientific) for sample acquisition using CytoFlex S flow cytometer (Beckman Coulter, Brea, CA, USA) and the data was analyzed using FlowJo V10 (Becton, Dickinson and Company, Franklin Lakes, NJ, USA). The gating strategy is available in Supplementary Figure S6.

### 4.6. Simple Western Jess Automated Protein Analysis

Detections of ARG1 protein in cells and EVs were carried out using the Protein Simple’s Jess capillary protein detection system. Cell and EV samples were lysed in RIPA buffer (ThermoFisher Scientific) supplemented with protease/phosphatase inhibitor (ThermoFisher Scientific), quantified using the BCA assay (ThermoFisher Scientific) and run for detection. To detect the engineered ARG1 protein, the separation module 12–230 kDa was used following manufacturer protocol. Briefly, 1 µg of sample and protein standard were run in each capillary, probed with primary antibodies followed by secondary antibodies provided in Jess kits (HRP/IR). Primary antibodies used are as following: rabbit anti-human ARG1 (ThermoFisher Scientific, clone 24H4L3, 1:100 dilution), mouse anti-human actin (Novus Biologicals, Centennial, CO, USA, clone AC-15, 1:20 dilution), rabbit anti-human CD9 (Cell Signaling, clone D9O1A, 1:100 dilution), rabbit anti-human calnexin (Novus Biologicals, 1:100 dilution), mouse anti-human CD81 (Novus Biologicals, 1:10 dilution), rabbit anti-HSP60 (R&D system, Minneapolis, MN, USA, 1:100 dilution), rabbit anti-human GM130 (R&D system, 1:10 dilution).

### 4.7. Quantification of Recombinant ARG1 Protein by ELISA

Human ARG1 protein carried on the ARG1 EVs was quantified by an enzyme-linked immunosorbent assay (ELISA) using precoated ELISA plates (Invitrogen, Carlsbad, CA, USA) following the manufacturer’s instructions. Briefly, EV samples were lysed with RIPA buffer (ThermoFisher Scientific) supplemented with protease/phosphatase inhibitor (ThermoFisher Scientific) at 1:1 ratio for 30 min on ice. A total of 1 × 10^11^/mL or 1 × 10^10^/mL of lysed EVs were plated to antigen-coated wells together with a biotinylated detecting antibody and incubated at room temperature for 2 h on an orbital shaker (200 rpm). After the incubation, the lysates were removed and the wells were washed four times with 1X Wash Buffer, and the Streptavidin-HRP was added into the wells and incubated for 1 h on an orbital shaker (200 rpm). Wells were washed four times after the incubation and TMP substrate was added into the wells to allow the color to develop. The reactions were then stopped with Stop Solution once the color was developed and absorbances at 450 nm and 620 nm were recorded using a BioTeck Gen5 plate reader (Agilent, Santa Clara, CA, USA). The concentrations of ARG1 carried on ARG1 EVs were calculated based on the standard curve.

### 4.8. Arginase Activities of ARG1 EVs

Arginase activities of the ARG1 EVs were quantified using a colorimetric arginase assay kit (BioAssay System, Hayward, CA, USA). EV samples were lysed with lysis buffer containing 0.4% Triton X-100 (Millipore Sigma Aldrich, Burlington, MA, USA) supplemented with protease/phosphatase inhibitor (ThermoFisher Scientific) for 30 min on ice, and 1 × 10^11^/mL, 1 × 10^10^/mL, and 1 × 10^9^/mL of ARG1 EVs were tested following the manufacturer’s instructions. Briefly, arginine substrate containing Mn were incubated with the samples for 2 h at 37 °C and the amount of urea produced by the enzymatic activities of arginase were detected using Urea Reagent by incubating for 1 h at room temperature. After incubation, optical density at 430 nm of the samples were measured using a BioTeck Gen5 plate reader (Agilent) and the arginase activities in the samples were calculated based on the urea standard.

### 4.9. In Vitro Delivery of ARG1 Protein by ARG1 EVs

To evaluate the capability and efficacy of EVs carrying ARG1 in delivering ARG1 protein into the cells, HepG2 cells were treated with either ARG1 EVs or recombinant human ARG1 (rHuArg1) (ACROBiosystems, Newark, DE, USA) for 6, 24, and 48 h, and the urea concentration in the culture supernatant and arginase activities in the cell lysate were measured. 1 × 10^5^ cells were seeded in 12 wells plate 1 day prior to the experiment to allow the cells to attach. To test the in vitro delivery of ARG1 by EVs, Arg1-out or Arg1-in EVs were diluted in 1X PBS to desired concentration ranging between 1 × 10^12^ and 1 × 10^11^/mL. For the rHuArg1, the protein was reconstituted in distilled water at 0.5 mg/mL and stored at −80 °C before the experiment without repeated free/thaw cycle. Similar to ARG1 EVs, rHuArg1 was further diluted to 1.25–0.0087 µg/mL using 1X PBS. Cells were washed once with EMEM/10% FBS followed by the treatment of EVs or rHuArg1. Cells treated with 1X PBS served as the negative control. To ensure the culture condition of the cells, the volume of EVs, recombinant protein, and PBS were 10% of the culture media. For each time point, the culture supernatant was collected and centrifuged at 1000× *g* at 4 °C to remove the debris before testing the urea concentration using a Urea Assay Kit (BioAssay System) by following the manufacturer’s protocols. Cells were kept on ice and washed once with cold 1X PBS followed by lysing with the lysis buffer containing 0.4% Triton X-100 (Millipore Sigma Aldrich) supplemented with protease/phosphatase inhibitor (ThermoFisher Scientific) for 30 min on ice. After incubation, cell lysates were centrifuged at 14,000× *g* at 4 °C and the supernatants were tested for their arginase activities by arginase activity assay (BioAssay System) by following the manufacturer’s instructions.

### 4.10. Exosome Labeling

EVs were labeled with IVISense 750 MAL Fluorescent Self-Quenching Dye (Revvity Health Sciences, Waktham, MA, USA). Briefly, 1 mL of 1 × 10^12^ particle/mL was incubated with 20 µg dye and incubated at 37 °C for 4 h. Free, unbound dye was removed using the Zeba Spin Desalting Columns (Thermofisher Scientific) according to the manufacturer’s instructions.

### 4.11. Animal Study—Biodistribution in Wild-Type Mice

Studies were conducted according to the guidelines of the Institutional Animal Care and Use Committee (IACUC protocol EB17-004-091, approved 08SEP2023). Mice were fed ad libitum and sterile water; housed in groups of five at 22 °C/30% humidity and light cycles of 0600–1800 h with standard nesting material; and allowed free movement. To examine the tissue distribution of ARG1 EVs, age-matched BALB/c mice (female, 8–10 wk old) received intraperitoneal injections (100 µL) of either (1) PBS, (2) 293F or (3) STX-Arg1-in EVs. A total of 1 × 10^11^ particles/100 µL were injected and blood and tissues (salivary glands, brain, lungs, heart, diaphragm, liver, spleen, kidney, lower limbs) were collected at 1 h, 24 h and 1 wk after injection. Full body and tissue imaging was acquired on the IVIS imager (PerkinElmer, Waltham, MA, USA). Additionally, blood clearance and liver accumulation were evaluated at shorter timepoints (5, 15, 30, 60 min after injection).

### 4.12. Animal Study—Toxicity in Wild-Type Mice

Studies were conducted according to the guideline of the Institutional Animal Care and Use Committee (IACUC protocol EB17-004-091, approved 08SEP2023). Mice were fed ad libitum and sterile water; housed in groups of five at 22 °C/30% humidity and light cycles of 0600–1800 h with standard nesting material; and allowed free movement. To address activity and evaluate toxicity of STX-Arg1 EVs repeated injections, age-matched BALB/c mice (female, 8–10 wk old) received intraperitoneal injections (100 µL) of either (1) PBS, or (2) STX-Arg1-in EVs, at the highest dose allowed by our manufacturing scale (0.012 mg/kg). Mice were divided into two dose groups: (1) Dose 1 received one i.p. injection per week, and (2) Dose 2 received two i.p. injections per week. Weight was monitored weekly. At the end of the fourth week, mice were anesthetized using isoflurane, blood collected from the submandibular vein (in EDTA coated tubes), and peripheral tissues (salivary glands, brain, lungs, heart, diaphragm, liver, spleen, kidney, lower limbs) processed for histological analysis. Blood was further processed for plasma isolation after centrifugation at 4000 rpm for 5 min at 4 °C. Plasma was analyzed for arginine levels (LSBio, Seattle, WA, USA, see below), as indirect measurements of STX-Arg1-in activity. Liver toxicity was additionally assessed by the quantification of aspartate aminotransferase (AST) (Abcam) levels in blood.

### 4.13. Animal Study—Efficacy in Arg1 Knock-Out Mice

Studies were conducted according to the guideline of the Institutional Animal Care and Use Committee (IACUC protocol EB17-004-091, approved 08SEP2023). Mice were fed ad libitum and sterile water; housed in groups of five at 22 °C/30% humidity and light cycles of 0600–1800 h with standard nesting material; and allowed free movement. To evaluate the therapeutic potential of STX-Arg1 EVs, Arg1 knock-out mice (B6.129-Arg1tm1Rki/J, Strain #:007741; RRID: IMSR_JAX:007741) were acquired from Jackson Laboratory. Colony was expanded from cryo-embryos to obtain heterozygous mice for subsequent breeding. Heterozygote mice are viable and fertile, while homozygous Arg1 mutant completely lack hepatic ARG1 activity, exhibit hyperargininemia, severe symptoms of hyperammonemia (including decerebrate posture, lethargy, and high-frequency tremor of the extremities, particularly the tail) and die between 10 and 14 days after birth [36,37]. Mice received either an i.p. injection of 10 µL/g every 2 or 4 days for a final dose of 30 µg/kg ARG1 as delivered by STX-Arg1-in EVs. Weight gain and growth was monitored across the study and lifespan recorded. Additionally, a time course study was performed to evaluate the distribution of STX-Arg1-in EVs in the liver of knock-out mice and its activity. After injection, the blood and the liver were collected at 2, 6 and 24 h after injection. The liver was lysed in RIPA buffer (ThermoFisher Scientific) with 1X Protease inhibitors (ThermoFisher Scientific). ARG1 levels were quantified by human ARG1 ELISA (Invitrogen). Blood was centrifuged at 4500× *g* for 10 min and plasma collected for arginine levels quantification by ELISA (LSBio, see below).

### 4.14. Arginine ELISA

Arginine levels in the blood were quantified by competitive ELISA (LSBio, Seattle, WA, USA) as per the manufacturer’s instructions. ELISA was used as an indirect measure of the STX-Arg1-in EVs in vivo. Briefly, 50 μL of either standards or samples are added to the wells together to a fixed quantity of biotin-conjugated target antigen. The antigens in the standards or samples compete with the biotin-conjugated antigen to bind to the captured antibody. Unbound antigen is washed away. An Avidin-Horseradish Peroxidase (HRP) conjugate is then added, which binds to the biotin. After the removal of unbound HRP-conjugate, a TMB substrate is then added, which reacts with the HRP enzyme resulting in color development. The reaction is topped with a sulfuric acid stop solution and the optical density (OD) of the well is measured at a wavelength of 450 nm: the greater the amount of antigen in the sample, the lower the color development and optical density reading.

### 4.15. Statistics

For in vitro studies of the delivery of ARG1 by EVs, the differences between ARG1 EVs and the rHuArg1 were tested using ordinary one-way analysis of variance (ANOVA) with Tukey’s multiple comparison test with a pooled variance to compare the urea concentration in the culture supernatants and the increase in arginase activities in the cell lysate between the HepG2 cells treated with different EVs or rHuArg1 at the same time points. A *p* value < 0.05 was considered statistically significant. For in vivo studies, a 2-tailed *t*-test or one-way ANOVA controlled for multiple comparisons were used. Details are to be found in figure legends. All the statistical analyses were performed using GraphPad Prism Version 10 (San Diego, CA, USA).

## Figures and Tables

**Table 1:** Summary of STX-Arg1 EV characteristics.

	EVs per 1 × 10^6^ Cells ^d^	Concentration (EVs/mL)	Size (nm)	PDI	Total Protein Concentration ^a^ (mg/mL)	ARG1 Concentration ^b^ (ng/mL)	Arginase Activity ^c^ (U/L)
Arg1-free	1.19 × 10^10^	0.96 × 10^12^	129.5 ± 49.8	0.148	0.027	3.7 ± 0.4	6.0 ± 0.0
Arg1-out	8.33 × 10^9^ ± 3.17 × 10^9^	2.20 × 10^12^	129.4 ± 47.2	0.133	0.026	7.0 ± 0.5	51.1 ± 1.3
Arg1-in	1.15 × 10^10^ ± 5.21 × 10^9^	3.03 × 10^12^	120.3 ± 46.7	0.151	0.025	8.7 ± 0.2	226.4 ± 5.7

^a^: Total protein concentrations were shown in 1 × 10^11^ EVs/mL. ^b^: ARG1 concentrations were shown in 1 × 10^11^ EVs/mL. ^c^: ARG1 activities were shown in 1 × 10^11^ EVs/mL. ^d^: For comparison, 293F cells yield 6.02 × 10^9^ ± 1.47 × 10^9^ EVs.

## Data Availability

The original contributions presented in this study are included in the article/Supplementary Materials. Further inquiries can be directed to the corresponding author.

## Supplementary Materials

The following supporting information can be downloaded at https://www.mdpi.com/article/10.3390/ijms27093785/s1.
