# Supplementary material for: Extracellular Vesicles Delivered a Functional ARG1 Enzyme and Restored Its Activity in a Mouse Model of ARG1-D Resulting in Improved Lifespan
Source: Int J Mol Sci. 2026 Apr 24;27(9):3785. doi: 10.3390/ijms27093785 (PMC13164535; doi:10.3390/ijms27093785)
Supplement: Supplementary file 1 [file ijms-27-03785-s001.zip › ijms-4265372-supplementary.pdf]

Supplementary Figures.

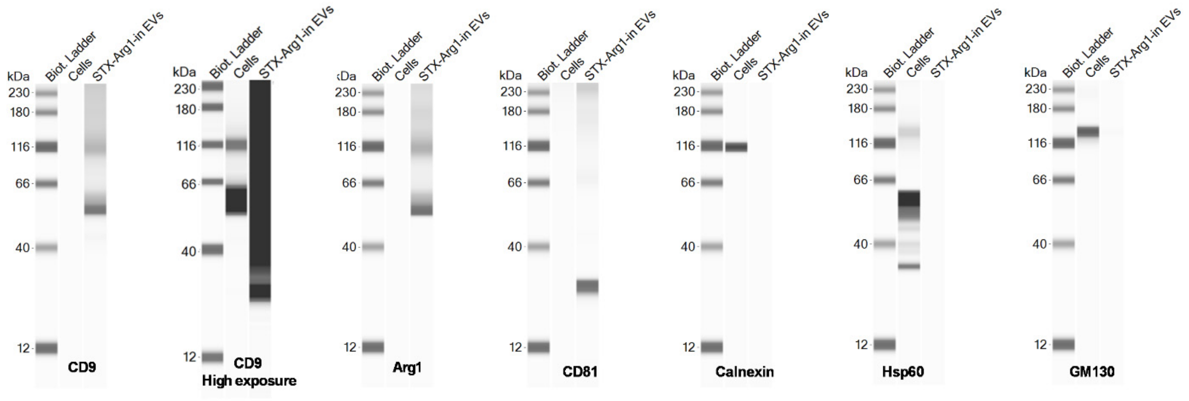

Supplementary Figure S1. Jess western blot.

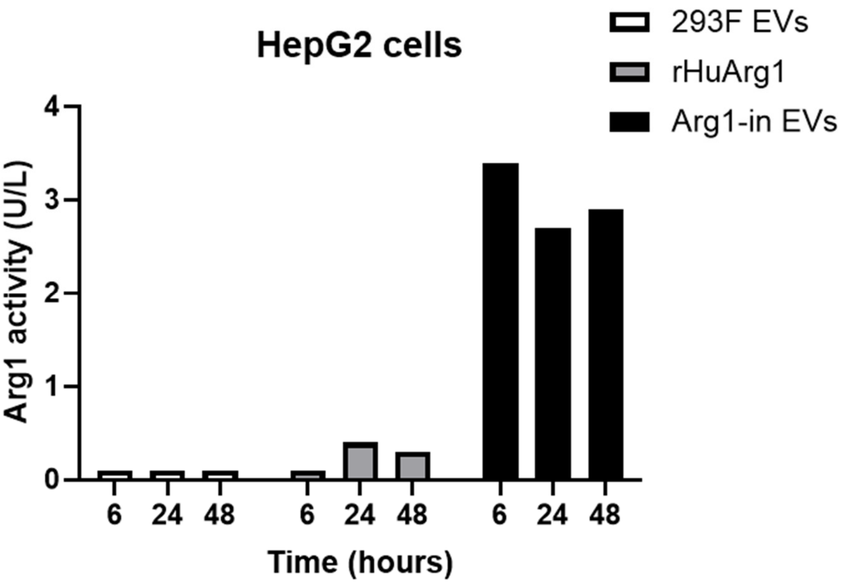

Supplementary Figure S2. 293F EVs have no biological effects in HepG2 cells.

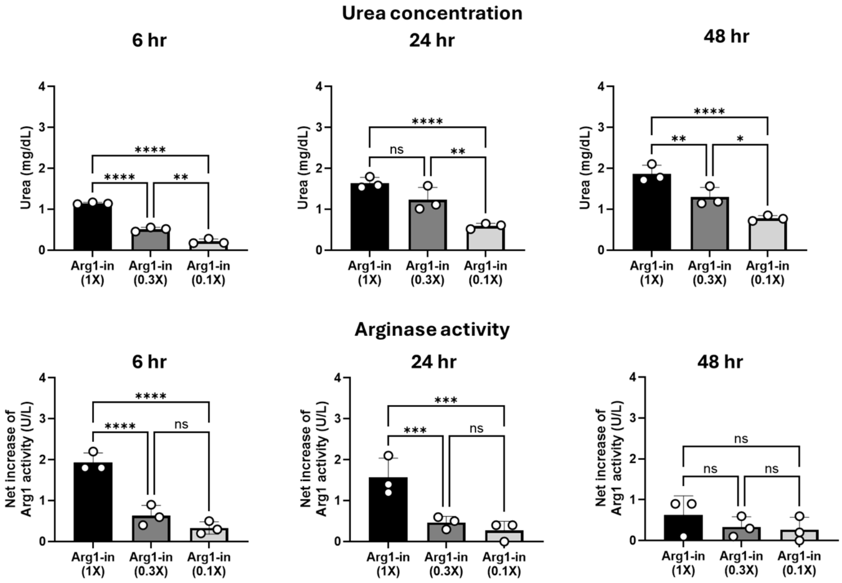

Supplementary Figure S3. Dose response of STX-Arg1-EVs: urea production and ARG1 activity

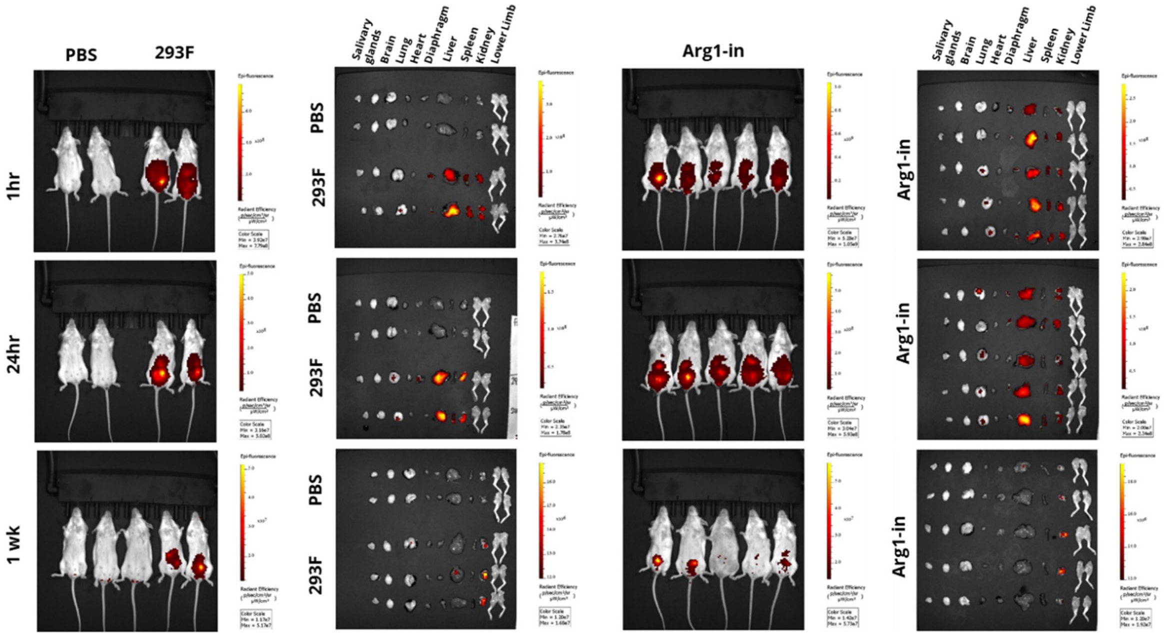

Supplementary Figure S4. In vivo imaging by IVIS imager for exosome biodistribution.

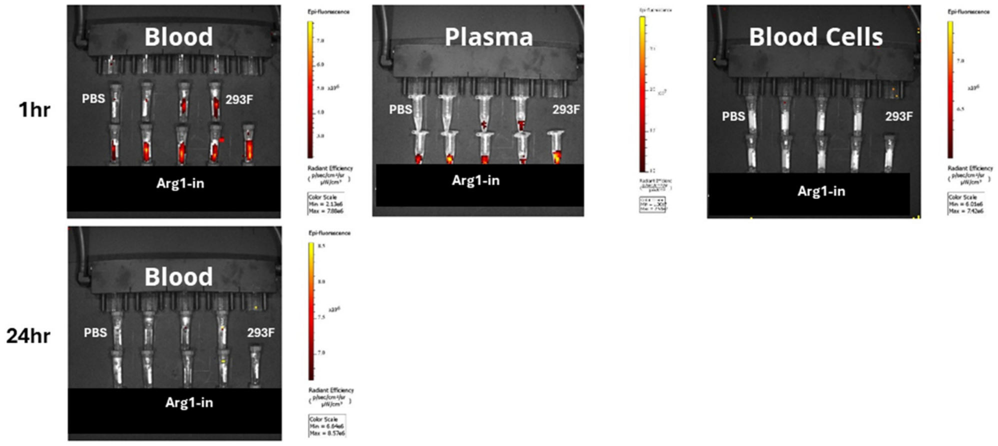

Supplementary Figure S5. IVIS imaging of blood.

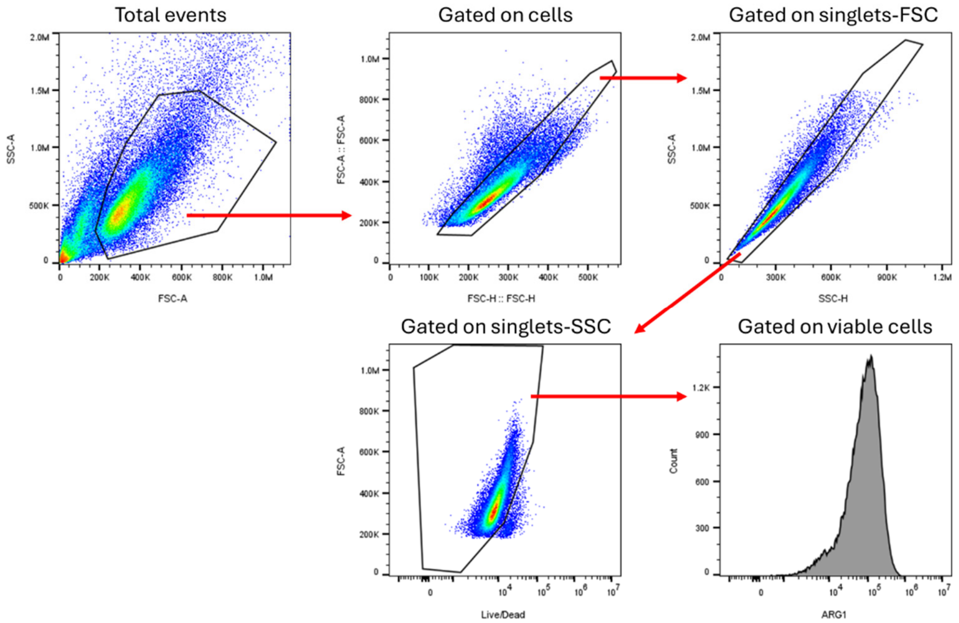

Supplementary Figure S6. Gating strategy to measure ARG1 expression of engineered cell lines
